# A single-nucleotide-polymorphism real-time PCR assay for genotyping of *Mycobacterium tuberculosis* complex in peri-urban Kampala

**DOI:** 10.1186/s12879-015-1121-7

**Published:** 2015-09-30

**Authors:** Eddie M. Wampande, Stavroula K. Hatzios, Beatrice Achan, Ezekiel Mupere, Mary Nsereko, Harriet K. Mayanja, Kathleen Eisenach, W Henry Boom, Sebastien Gagneux, Moses L. Joloba, For the Tuberculosis Research Unit

**Affiliations:** Department of Medical Microbiology, College of Health Sciences, School of Biomedical Sciences, Makerere University, P.O BOX 7072, Kampala, Uganda; Department of Pediatrics and Child Health College of Health Sciences, Makerere University, Kampala, Uganda; Uganda-Case Western Reserve University Research Collaboration, Kampala, Uganda; Department of Pathology, University of Arkansas for Medical Sciences, Little Rock, AR USA; Tuberculosis Research Unit, School of Medicine, Case Western Reserve University and University Hospitals of Cleveland, Cleveland, Ohio USA; Department of Medical Parasitology and Infection Biology, Swiss Tropical and Public Health Institute, Basel, Switzerland; University of Basel, Basel, Switzerland; Department of Bio-molecular Resources and Biolab Sciences, College of Veterinary Medicine, Animal Resources and Bio Security, Makerere University, Kampala, Uganda; Division of Infectious Diseases, Brigham and Women’s Hospital, Boston, Massachusetts, USA Department of Microbiology and Immunobiology, Harvard Medical School, Boston, MA USA

**Keywords:** Single nucleotide polymorphism, Lineages, Long sequence polymorphism, High-throughput

## Abstract

**Background:**

Accurate and high-throughput genotyping of *Mycobacterium tuberculosis* complex (MTBC) may be important for understanding the epidemiology and pathogenesis of tuberculosis (TB). In this study, we report the development of a LightCycler® real-time PCR single-nucleotide-polymorphism (LRPS) assay for the rapid determination of MTBC lineages/sublineages in minimally processed sputum samples from TB patients.

**Method:**

Genotyping analysis of 70 MTBC strains was performed using the Long Sequence Polymorphism-PCR (LSP-PCR) technique and the LRPS assay in parallel. For targeted sequencing, 9 MTBC isolates (three isolates per MTBC lineage) were analyzed for lineage-specific single nucleotide polymorphisms (SNPs) in the following three genes to verify LRPS results: Rv004c for MTB Uganda family, Rv2962 for MTB lineage 4, and Rv0129c for MTB lineage 3. The MTBC lineages present in 300 smear-positive sputum samples were then determined by the validated LRPS method without prior culturing.

**Results:**

The LSP-PCR and LRPS assays produced consistent genotyping data for all 70 MTBC strains; however, the LSP-PCR assay was 10-fold less sensitive than the LRPS method and required higher DNA concentrations to successfully characterize the MTBC lineage of certain samples. Targeted sequencing of genes containing lineage-specific SNPs was 100 % concordant with the genotyping results and provided further validation of the LRPS assay. Of the 300 sputum samples analyzed, 58 % contained MTBC from the MTBC-Uganda family, 27 % from the MTBC lineage 4 (excluding MTBC Uganda family), 13 % from the MTBC lineage 3, and the remaining 2 % were of indeterminate lineage.

**Conclusion:**

The LRPS assay is a sensitive, high-throughput technique with potential application to routine genotyping of MTBC in sputum samples from TB patients.

**Electronic supplementary material:**

The online version of this article (doi:10.1186/s12879-015-1121-7) contains supplementary material, which is available to authorized users.

## Introduction

*Mycobacterium tuberculosis* (MTB) is an acid-fast bacillus that causes tuberculosis (TB) a chronically debilitating disease with a mortality rate approaching 2 million deaths per year [1–3]. The disease primarily develops in 5–10 % individuals following inhalation of air droplets containing *Mycobacterium tuberculosis complex* (MTBC) bacilli, but may also occur following reactivation of a latent infection [4]. In Kampala, Uganda, 3 dominant MTBC genotypes have been identified namely MTBC Uganda family that accounts for 63 % of TB cases, followed by other MTBC lineage 4 genotypes other than Uganda genotype and then MTBC lineage 3 [5, 6]. These genotypes present with diverse clinical outcomes for instance MTBC Uganda family genotypes are less prone to drug-resistance, less virulent, and not associated with extra pulmonary TB [5, 7–10]. The MTBC lineage 4 genotypes progress rapid to disease compared to other genotypes [11, 12], while the MTBC lineage 3 genotypes cause severe disease [13]. Therefore accurate determination of the MTBC strain diversity within a population like Kampala can lead to the design of intervention strategies that more effectively target circulating strains.

The currently available MTBC genotyping assays are challenging to implement in areas with endemic TB and are limited in their ability to discriminate MTBC strains present in clinical isolates. For example robust techniques such multi-locus sequence typing (MLST) [14] and whole genome sequencing (WGS) [15, 16], are difficult to adopt in resource-limited countries because they are prohibitively expensive [17]. Other techniques, such as MIRU-VNTR, IS6110-RFLP, PGRS-RFLP, and CRISP [18, 19], can erroneously classify MTBC lineages [16, 20] due to homoplasy and are technically cumbersome. Furthermore, some of these methods typically require prior culturing of MTB from sputum samples, a process that takes 1–2 months [21]. For samples containing a mixed MTBC population, this culturing step may skew strain diversity by promoting growth competition between different strains [22]. Thus, there is a need for a more robust genotyping assay that is fast, sensitive, and can be applied directly to processed sputum samples without prior culturing.

To mitigate the aforementioned flaws a real-time PCR (RT-PCR) assay—the **L**ightCycler® 480 **R**T-**P**CR **S**NP (LRPS) assay—was developed to genotype MTBC directly from processed sputum samples using hybridization probes. This assay was evaluated for the ability to accurately identify MTBC lineages in peri-urban Kampala and subsequently used to analyze 300 smear-positive sputum samples from individual patients.

## Materials and methods

### Identification of lineage-specific SNPs for genotyping MTBC

The MTBC lineage-specific SNPs used in this study were obtained from whole genome sequencing data as previously described [14 16] with reference to the first MTBC (i.e., H37Rv) genome [23] to be sequenced. A SNP corresponding to a specific MTB lineage/sublineage was annotated by showing its position in the corresponding gene (ORF) and the associated nucleotide change (See Additional file 1: Table S1).

### Design of primers and probes for LRPS assay

Primers and probes for typing MTBC Uganda family (MTB L4-U) MTBC lineage 4 excluding the MTBC Uganda family (MTB L4-NU), and MTBC lineage 3 (MTB L3) were chosen based on the list of lineage-specific SNPs described previously [14, 16] (See Additional file 2: Table S2). LightCycler® Probe Design Software 2 (Roche Applied Science, Germany) was used for the design of assay primers and probes. In brief, RT-PCR enables the quantitative detection of a particular segment of DNA by coupling a fluorescent signal with DNA amplification. The fluorescence produced during amplification is directly proportional to the amount of DNA present in a given sample, amplification efficiency of the primer and probe combination. In order to distinguish the DNA of different MTBC lineages, hybridization probes were designed to recognize unique SNPs that are specific to particular MTBC lineages/sublineages. To identify MTBC L4-U and MTBC L3, hybridization probes were designed to perfectly complement wild type (H37Rv) DNA for MTBC L4-NU probes were designed to complement the mutant DNA. Thus, for MTBC L4-U and MTBC L3 probes will produce lower melting temperature (T_m_) values (due to single base mismatch) than samples with wild type DNA (due to perfect match), whereas the MTBC L4-NU probes will produce higher T_m_ values with mutant DNA (due to perfect match). We used MTBC lineage 3 (CAS), Uganda family and H37Rv DNA as controls.

### MTBC DNA extraction

The genomic DNA from stored isolates was extracted as described by Wampande et al. Stucki et al. [5, 24]. Frozen isolates that were earlier characterized as MTBC by IS6110-PCR were thawed, centrifuged, and the pellet washed twice with phosphate buffer saline (PBS). Pellets were subsequently reconstituted in 100 μl PCR water, heated at 90 °C for 1 h, and sonicated for 15 min to completely lyse the bacilli and release the genomic DNA. The latter was recovered in the supernatant following centrifugation at 13000 g for 30 min, quantified by Qubit® 2.0 fluorometer (Invitrogen, USA) and used immediately or stored at −20 °C for future use.

### Long sequence polymorphism (LSP) -PCR analysis

In order to ascertain whether the LRPS assay correctly identifies MTBC lineage we compared the LRPS results with LSP-PCR data using genomic DNA extracted from 70 MTBC stored isolates. LSP-PCR was performed using RD 724 deletion primers (specific for MTB Uganda family) and RD750 deletion primers (Specific for MTB lineage 3) as described by Gagneux et al. [25] and Tsolaki et al. [26]. The 10 μl reaction volume PCR was containing 5.5 μl water, 1 μl (10 μM final concentration) forward primer (RD 724 or RD 750) and 1 μl (10 μM final concentration) reverse primer (Reverse RD 724 or RD 750), 1 μl of 10 x Thermo Fischer Scientific Custom PCR Master mix, 1 μl template DNA (at least 50 ng) and 0.5 μl (0.5 unit) DNA polymerase. The reaction was run in a standard thermocycler programmed at 95 °C for 10 min 35 cycles of 95 °C for 1 min, 64 °C for 30 s and 72 °C for 30 s. PCR products were analyzed by gel electrophoresis.

### PCR and targeted sequencing

To further validate the LRPS assay target sequencing of three ORFs (Rv004c for MTBC L4-U Rv2962 for MTBC L4 and Rv0129c for MTBC L3) which contain lineage-specific SNPs from 9 MTBC isolates was performed using primers in Table 1. The PCR was run in a 20 μl reaction volume with 12 μl water 1 μl forward primer and 1 μl reverse primer (0.5 μM of each of the primers), 4 μl of 5 x Roche genotyping master mix (containing Taq polymerase and dNTPs), and 2 μl template DNA (at least 50 ng). The reaction was run in a standard thermocycler (95 °C for 10 min 35 cycles of 95 °C for 10 s primer(s) annealing (57 °C for Rv004c ORF or 53 °C for Rv0129c ORF or 51 °C for Rv2962 ORF) for 10 s 72 °C for 10 s. PCR products were analyzed by gel electrophoresis purified by Qiagen PCR purification kit and commercially sequenced using the primers designed for the specific ORFs (See Table 1). To confirm the presence of the SNP in the respective ORFs, BioEdit software (Ibis Biosciences, USA) was used to align the corresponding H37Rv ORF with the sequenced fragments.

**Table 1:** SNPs markers, primers and probes used in RT-PCR

SNP Name	Primer and probes	Tm-1	Tm-2	Primer annealing temperature	Fragment length (bp)
Rv004c-0619n (MTB L4-U)	Forward:5-ATT GCT CGA TGG CAG A-3	^a^62 °C	68 °C	57 °C	160
	Reverse: 5-AAA CCA GGT ACT TGT CGG-3				
	LC Red 640-TGA TGA CGG AAA GCC GT GAA A-Pho-3				
	5-GTT TTC GCG GTA GGT GCC CTC GAT G-Fluo-3				
Rv2962c-0711 s (MTB L4-NU)	Forward: 5-GAA CGC CCT TTG CTC TTC-3	56 °C	^a^64 °C	51 °C	181
	Reverse:5-CAA GGT ACT CGT GGT TGG-3				
	LC Red 610- CCC GAG CTG ATG CCC ACC T-Pho-3				
	5-CAC ACC CTG TAT GC GAC G-Fluo-3				
Rv0129c-0472n (MTB L3)	Forward:5-CGA CTG GTA TCA GCC CTC-3	^a^58 °C	68 °C	53 ° C	191
	Reverse:5-GGA ACT GCT GCG GGT AGT A-3				
	LC Red 610-GAC ACG CC TTG TTG GCC-Pho-3				
	5-CGC CGC GTT GCC TGT CG –Fluo-3				

Shaded and bold nucleotide denotes SNP position relative to H37Rv MTB genotype

^a^Denotes MTB lineage specific melting temperature (Tm)

### Patient recruitment and processing sputum samples

Patients were recruited from Mulago hospital TB clinic (ward 5 & 6) which serves as the main referral TB centre in Uganda. Sample processing confirmatory microscopy (both ZN and auramine staining) and culturing was done at Mycobacteriology Laboratory, Department of Medical Microbiology, College of Health Sciences, Makerere University. 300 sputum samples were processed in a Biosafety cabinet class II following standard procedures [27]. The final sediment was suspended in 2.5 ml PBS buffer (pH 6.8) part of this sample was used to inoculate Middlebrook 7H10 supplemented with 10 % (*v/v*) glycerol and OADC for culturing following standard procedures [28] and the remainder was re-suspended in 50 μl of PCR water for DNA extraction. The latter was heat killed at 95 °C for 1 h and later sonicated for 15 min to completely lyse the bacilli and release the genomic DNA. The genomic DNA was obtained in the supernatant following centrifugation at 13000 rpm and stored at −20 °C or used immediately in the LRPS assay.

### LRPS genotyping assay

Genotyping of MTBC from cultured and frozen isolates or processed sputum samples was performed using ORF-specific primers and probes designed based on SNP positions (See Table 1 and Additional file 2: Table S2) in Roche LightCycler ® RT-PCR 480 machine (Roche Applied Science Germany). Briefly, the LRPS assay was run in 20 μl reaction containing 11.2 μl of PCR water, 1 μl (0.5 μM final concentration) reverse primer, 1 μl (0.5 μM final concentration) of forward primer, 0.4 μl (0.4 μM final concentration) of the donor probe, 0.4 μl (0.4 μM final concentration) of the acceptor probe, 4 μl of 5 x Roche genotyping master mix, and 2 μl (containing at least 5–50 ng) of extracted genomic DNA. The Roche LightCycler® 480 (Roche Applied Science, Germany) was programmed for PCR amplification and a melting curve stage. For each of the three uniplex assays, the amplification stage consisted of a pre-PCR stage performed at 95 °C for 10 min, an amplification stage with denaturation at 95 °C for 10 s, primer annealing (57 °C for Rv004c ORF or 53 °C for Rv0129c ORF or 51 °C for Rv2962 ORF) for 10 s with a single acquisition mode to allow capture of the fluorescence, and extension at 72 °C for 10 s for 45 cycles. The melting curve analysis consisted of denaturation of amplicons at 95 °C for 1 min to produce single stranded DNA (ssDNA), probe annealing temperature at 40 °C (allows hybridization of the probes to the complimentary sites of the ssDNA) for 10 s, probe melting temperature ranging from 40–80 °C (allows the probe to detach from the ssDNA) with a continuous mode of acquisition at a rate of 1 acquisition/s that allows capture of the fluorescence.

### Data analysis

From the melting curves the LightCycler® 480 software version 1.2 (Roche Applied Science, Germany) was used to derive probe melting temperature (Tm), which is lineage-specific. For MTBC Uganda family and MTB Lineage 3, the Tm was lower (due to mismatch) than the wild type (perfect match) yet for MTBC lineage 4 the Tm (due to perfect match) was higher than the wild type.

### Ethical consideration

This study obtained ethical approval from Makerere University institutional review board and the Uganda National Council for Science and Technology. Written informed consent was obtained from all the study participants.

## Results

### Primers and probes used in genotyping MTBC

A total of eight primer/probe sets were successfully designed using the LightCycler® probe design (Roche Applied Science Germany) software 2 (Additional file 2: Table S2). During the LRPS optimization step primer probe set Rv0006^a^ and Rv0407^b^ failed to give signals (no amplification), primer/probe set Rv3133c^C^ and Rv2959c were giving results that were conflicting with the positive (Central Asian Strain, CAS) and negative controls (H37Rv), thus these sets were excluded. Also Primer/probe set Rv2949c^a^ was excluded from further sample analysis since it required twice the probe concentration as the counterpart. Therefore primer/probe sets Rv004c^a^ was used to analyze samples for presence of MTBC Uganda family; Rv2962^b^ and Rv0129c^c^ primer sets were used to identify MTBC lineage 4 and MTBC lineage 3 respectively (Table 1).

### Identification of MTBC Uganda family using LSP-PCR and LRPS assay

**Fig. 1:**
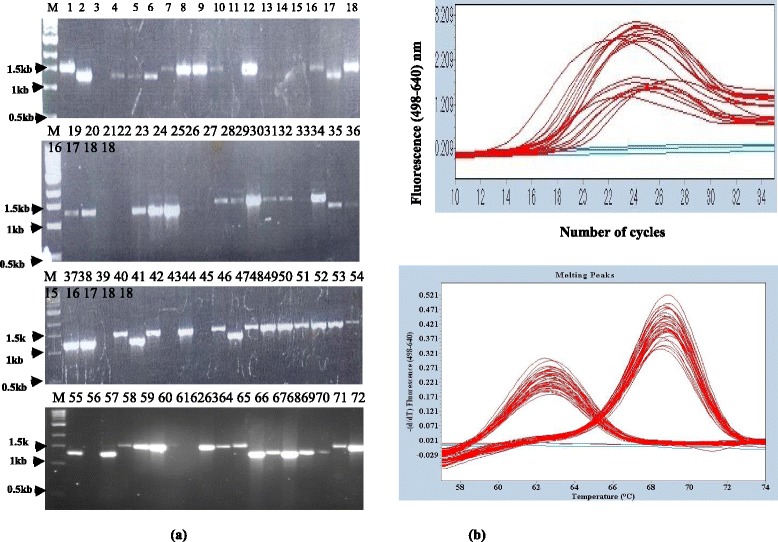
Comparing RD 724 LSP-PCR and LRPS typing: Samples (*N* = 70) were analyzed by primers specific for RD 724 (lane M = DNA marker, 1 = MTB Uganda genotype, 2 = H37Rv, 3 = negative control (no DNA added), lane 4–72 = test samples) a band of 1.5 kb identifies MTB Uganda family, while that of 1.3 kb identifies other MTBC other than Uganda family (Panel **a**). For Panel **b** specific primers/probes (Rv004c primer/probe set) containing lineage specific SNP were used, the *top panel* shows the amplification (see also Additional file 5: Figure S2a) of the target region while the *bottom panel* shows the derived melting temperature. A peak of 62 °C shows the Uganda family while that of 68 °C shows the wild type (non-Uganda family)

LSP-PCR and targeted sequencing reactions were run in parallel to validate the LRPS assay. A total of 70 MTBC isolates (confirmed by IS6110 PCR) were genotyped using LSP-PCR with primers specific for the RD 724 deletion in parallel with LRPS assay using Rv004c^a^ primer/probes set (Table 1). While both assays were equally capable of identifying MTBC Uganda (Fig. 1 and Additional file 5 Fig S2(a)) only the LRPS assay was able to genotype all 70 samples the LSP-PCR assay failed to identify the MTBC lineages of several isolates (Fig. 1: lane 11 13, 14, 15, 21, 22, 26, 27, 33, 39, 43, 45, 56, 61, 62). With that result, both assays were re-evaluated using serially diluted H37Rv genomic DNA (10 ng–100 ng for LSP-PCR and 1 ng–10 ng for LRPS per PCR reaction) extracted by the Enzyme/CTAB method [29]. The detection limit of the LSP-PCR assay was 10-fold higher than that of the LRPS assay (30 ng approximately 7 × 10^6^ copies/reaction and 3 ng approximately 7 × 10^5^ copies/reaction) respectively (Additional file 4: Figure S1). Later, DNA from those samples that previously produced no LSP-PCR product (Fig. 1: lane 11,13,14,15, 21, 22, 26, 27, 33, 39, 43, 45, 56, 61, 62) were re-extracted and repeated the LSP-PCR genotyping reactions with at least 30 ng template DNA. This time, genotyping data were obtained for all isolates and were in agreement with those of the LRPS assay (data not shown).

### Identification of MTBC Lineage 3 genotype using LSP-PCR and LRPS

**Fig. 2:**
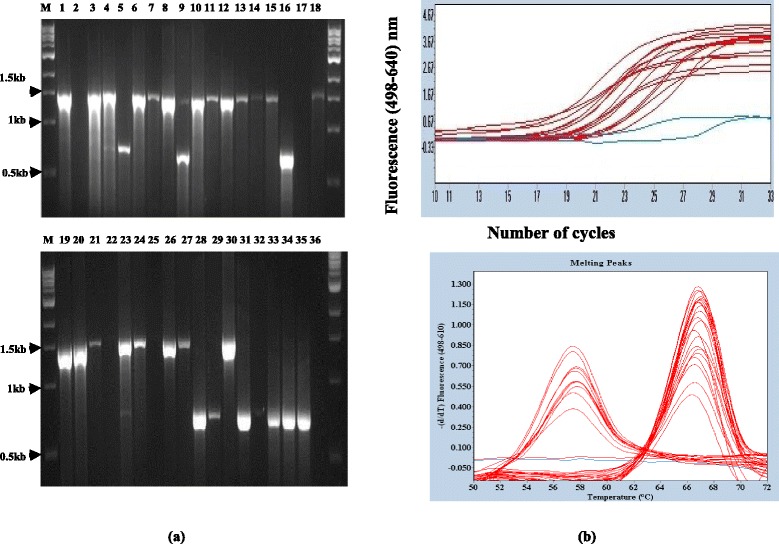
Comparing RD 750 LSP-PCR and LRPS typing: Samples (*N* = 33) were analyzed by primers specific for RD 750 (lane M = DNA marker, 1 = H37Rv, 2 = negative control (no DNA added), lane 3–36 = test samples), a band of 0.75 kb identifies MTB Lineage 3, while that of 1.3 kb identifies other MTBC other than MTB Lineage 3 (See Panel **a**). For Panel **b** specific primers and probes (Rv0129c^c^ primer/probe set) containing Lineage-3 specific SNP were used, the *top panel* shows the amplification (see also Additional file 5: Figure S2) of the target region while the *bottom panel* shows the derived melting temperature. A peak of 57 °C shows MTB lineage 3 while that of 67 °C shows the wild type (non-lineage 3)

A total of 33 MTBC isolates (See Additional file 3: Table S3) that were not MTBC Uganda (non-Uganda family MTBC) by RD 724 analysis (See Fig. 1 lane 4 5, 6, 17, 19, 20, 23, 24, 25, 35, 36, 37, 38, 41, 47, 57, 65, 66, 67, 68, 69 plus 12 isolates that were initially negative by RD724 analysis) were further genotyped by LSP-PCR (RD 750 deletion) and LRPS using MTBC L3-specific probes (Rv0129c^c^ primer/probe set). Both assays were 100 % concordant in identifying MTBC lineages 3 (Fig. 2a lane 5 9, 16, 28, 29, 31, 32, 33, 34, 35, Fig. 2b peak with 57 °C and Additional file 5 Fig S2b).

### Sequencing PCR products to identify MTB lineage-specific SNP

**Fig. 3:**
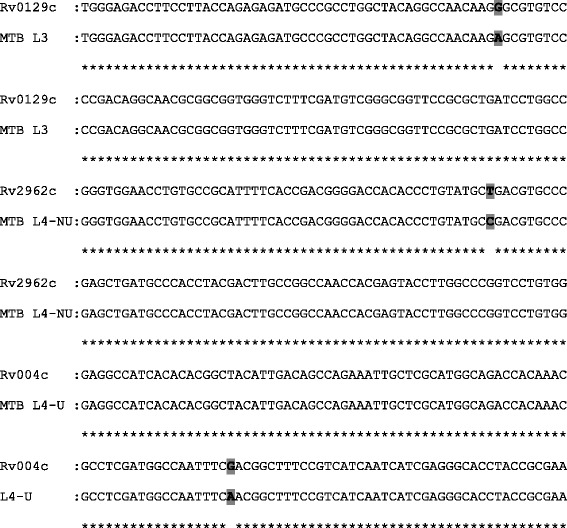
Pair wise alignment of H37Rv (wild type) and MTB lineage (mutant) sequences: BioEdit Version 7.2.5 (Ibis Biosciences, USA)was used to align the wild type (Rv004c, Rv2962 and Rv0129c) and the corresponding mutant (MTB L3, MTB L4-NU or MTB L4-U) sequences. The *shaded* and *bold* nucleotide shows the point mutation

To ascertain the accuracy of LRPS in genotyping MTBC lineages PCR products of 9 MTBC isolates (3 isolates for each lineage) were sequenced. The resulting sequences of the gene containing the lineage-specific SNP were compared with the corresponding H37Rv sequences using Bio Edit software (Ibis Biosciences, USA) the data confirmed that the sequenced PCR products contained lineage-specific SNPs (See Fig. 3).

### LRPS typing directly from processed sputum samples

**Fig. 4:**
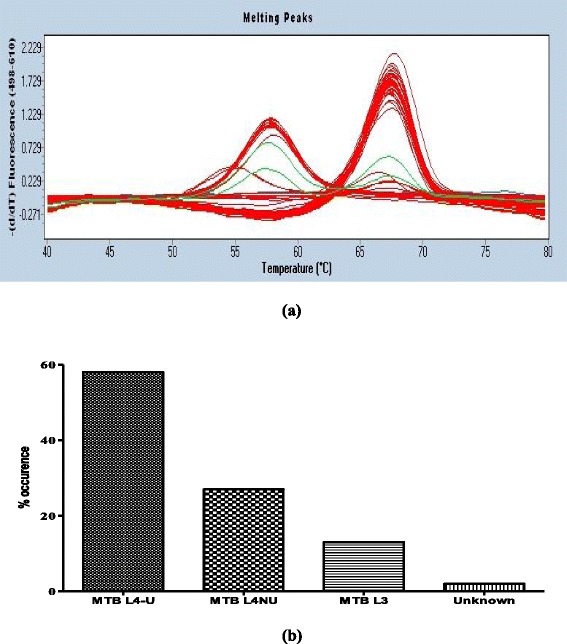
Analysis of smear positive sputum samples: LRPS identified MTB lineages/sublineages in the sputum samples (*N* = 300). Panel **a** shows the derived melting curves showing single genotypes (*Red*; single peaks) and double genotypes (*Green*; double peaks). Panel **b** shows the overall proportion of MTBC lineages from the total number of samples analyzed

A total of 300 freshly processed sputum samples were analyzed using LRPS (with primer/probe set Rv004^a^ for MTBC L4 U Rv2962^b^ for MTBC L4-NU and Rv0129c^c^ for MTBC L3). Of those 58 % (174/300) were MTBC L4-U, 27 % (82/300) MTBC L4-NU, 13 % MTBC L3 (39/300) and 2 % (5/300) could not be classified (See Fig. 4b) 4 % (11/300) had more than one genotype (double peaks in green) of MTBC. Efforts to confirm the presence of more than one genotype (for samples with double peaks) in the sputum sample by 15 MIRU-VNTR were futile since none of the alleles was amplifiable. These data corroborated well with the matched sputum samples that were cultured and later genotyped with LRPS except for samples that had more than one genotype (i.e. only one genotype was identified). For LRPS approximately 2 h are required to analyze 92 samples [the LightCycler® RT-PCR 480 machine uses 96 multi well sample plates) from the time of sample preparation to genotype identification.

## Discussion

A number of useful SNPs for robust genotyping of MTBC have been made available following the interrogation of whole genome sequence (WGS) data from global MTBC strains [14, 16, 30, 31]. Comparisons of SNPs with other markers used in molecular epidemiological studies of MTBC have proved their superiority since they are able to discriminate closely unrelated (no homoplasy) genotypes of MTBC [16]. Therefore, this study evaluated the use of novel single nucleotide polymorphic (SNP) markers in a LightCycler ® 480 (Roche, Germany) real-time PCR (LRPS) assay to genotype MTBC isolates using heat inactivated samples. First, SNP-pecific primers/probes were designed to accurately delineate MTBC lineages by real time PCR (RT-PCR). Secondly, the optimized and validated LRPS was used to sub-type MTBC lineages present in 300 smear-positive sputum samples from different individuals. By and large, these data suggest that LRPS can be used to accurately identify MTBC genotypes using heat killed crude lysates of processed sputum samples without prior culturing.

The findings show the successful use of 3 sets of primers/hybridization probes containing MTBC lineage-specific SNPs in RT-PCR (LRSP) to accurately delineate MTBC lineages (MTBC L4-U MTBC L4-NU and MTBC L3). The LRSP assay is based on coupling a fluorescent dye to an amplified segment of DNA and use of MTBC lineage specific probes-conjugated to dyes that ensures real-time identification of the genotype in 2 h for every 92 samples. This is contrary to other MTBC genotyping assays that require different step(s) for detection of the genotype, hence increasing the turnaround time 1–3 days depending on the method [17]. Comparison of LSP-PCR and targeted sequencing data of genes containing these lineage-specific SNPs as a step to validate the SNP assay indicate 100 % concordance. This agreement was not surprising since LSP-PCR, LRPS and sequencing methodologies have been used before to accurately genotype MTBC [14, 16, 32]. However, the detection limit of LSP-PCR was 10-fold higher than the SNP-based assay, thus rendering LRPS more sensitive (3 ng/assay or 7 × 10^5^ copies/reaction) enough to be used with low DNA concentration as well as with heat-inactivated samples. The advantage of using heat lysates eliminates the long steps in DNA extraction which is labor intensive and time consuming. Thus, the LRPS may be better suited for genotyping MTBC from processed sputum samples, which can have a low bacillary load. The enhanced ability to detect small amounts of DNA in a sample by LRPS can likely be attributed to the more sensitive fluorescence detection system of the Roche LightCycler® 480 (Roche Applied Science, Germany) machine [33]. In contrast, conventional PCR-based assays are limited by quantifiable DNA, the relatively poor sensitivity of gel-based DNA detection systems, and have a long turnaround time [34]. To support this observation, PCR-based genotyping methods such as spoligotyping and MIRU-VNTR require 20–50 ng of template DNA for a successful run if the detection system is modified as seen in luminex spoligotyping (Luminex Technology, TX, USA) or automated MIRU-VNTR, the sensitivity increases and the turnaround time is reduced, but these methods are still prone to misclassification of MTB lineages [17]. Taken together, this data suggest that the LRPS is more sensitive than the LSP-PCR approach and fast in identifying MTBC in clinical samples since the culturing step is eliminated, making it more suitable for early TB diagnosis, genotyping applications that involve samples with low bacillary loads for instance in TB/ HIV co-infected patients and smear negative TB patients.

Unlike the LRPS most available genotyping methods rely on prior culturing of MTBC, which is labor-intensive, time-consuming, and introduces the risk of selective growth in cases of mixed infections [35]. While previous efforts have been made to sub-type MTBC isolates directly from sputum using MTB lineage-specific PCR, MIRU-VNTR and spoligotyping, these approaches have seen limited success [35, 36] due to their requirement for relatively high amounts of DNA and/or the presence of inhibitors in the sample and a long turnaround time. In the current study, validated novel SNP-based genetic markers were evaluated to genotype MTBC isolates directly from processed sputum by LRPS without prior culturing. The assay successfully genotyped 300 MTBC isolates from sputum samples of these 58 % were classified as MTB L4-U, 27 % as MTB L4-NU, 13 % as MTB L3 and 2 % as unknown MTBC lineage, and these proportions did not significantly differ from the work published by Wampande et al., 2013 [6]. Notably, the LRPS assay was able to detect more than one genotype in certain isolates (11/300) in contrast the MIRU-VNTR method failed to reveal mixed infections in these samples, presumably due to the lack of sufficient DNA. Overall, these data indicate that the LRPS assay can be used directly on smear-positive, processed sputum samples to genotype MTBC. Due to its high sensitivity and the use of 2 probes with distinct melting curves, this assay has the potential to detect mixed infections in clinical isolates.

### Limitations

While the probes used in this study were designed to rapidly identify the three MTBC lineages circulating in peri-urban Kampala [5] additional MTBC lineage-specific probes would need to be developed to genotype other MTBC lineages/sublineages. The current assay is robust in defining deep phylogeny, but is alone not suitable for transmission studies in such circumstances MIRU-NVTR could be used in tandem with the LRPS assay. PCR inhibition was not observed in the LRPS assay, but, it could be relevant and impact negatively on the assay due to minimal buffering. Furthermore, only smear-positive sputum samples, which typically contain a high MTB DNA concentration were analyzed in this study thus further studies will be required to evaluate assay performance on smear-negative samples. The maintenance and initial cost of Roche LightCycler® 480 (Roche Applied Science, Germany) machines are very high, however LRPS is robust, of high throughput, and fast to perform: it has diverse applications, for instance, mRNA display studies, HIV viral load studies and as an ordinary PCR machine.

## Conclusion

The LRPS assay is a sensitive rapid, simple and high-throughput technique for detecting and/or genotyping MTBC from minimally processed, smear positive sputum and should be broadly applicable to genotyping SNPs in other microorganisms.

## Additional files

Additional file 1: Table S1. Lineage-specific SNPs for MTB Uganda family, MTB lineage 4 and MTB lineage 3. (DOCX 20 kb) Additional file 2: Table S2. Primer/probe used in genotyping MTB. (DOCX 21 kb) Additional file 3: Table S3. LSP-PCR and LRPS sample analyses. (DOC 43 kb) Additional file 4: Figure S1. LRPS is more sensitive than LSP-PCR typing: H37Rv genomic DNA was serially diluted and analyzed with RD 724 (*N* = 10) or RD 750 (*N* = 10) deletion primers: lane M = molecular weight markers, lane 1 positive control (MTB Uganda family or lineage 3 strain), lane 2 negative control, lane 3- 12 H37Rv DNA diluted from 100-10 ng; the minimum dilution of genomic DNA that can be amplified was in lane 10 (30ng) (Plate a & c). Plate b & d are the corresponding LRPS using Rv004c/probe or Rv0129c/probe set with H37Rv genomic DNA diluted from (10-1) ng per assay, the minimum dilution of DNA that can be amplified was 3ng (See arrow on the amplification curves plate b & d). (DOC 179 kb) Additional file 5: Figure S2. Fluorescence applification curve readings: Specific Rv004c and Rv0129c primer/probe set containing lineage specific SNP were used to analyze 70 and 33 MTBC isolates respectively. Amplification fluorescence curves for Rv004c and Rv0129 target region are shown in panel (a) and panel (b) respectively. (DOC 123 kb)

## Abbreviation

MTBC: *Mycobacterium tuberculosis* complex
TB: Tuberculosis
Tm: Melting temperature
SNP: ingle nucleotide polymorphism
RT-PCR: Real time polymerase chain reaction
LSP-PCR: Long sequence polymorphism-Polymerase chain reactiom
MLST: Multilocus sequence typing
RFLP: Restriction fragment length polymorphism
IS6110-PCR: Insertion sequence 6110-Polymerase chain reaction
MIRU-VNTR: Mycobacterial interspaced Repetitive Units- variable number of tandem repeats
ORF: Open reading frame
PGRS: Polymorphic Guanine cytosine-rich repetitive sequence
CRISPR: Clustered regularly interspaced short palindromic repeats)
NALC/NaOH: N-acetyl-L-cysteine-sodium hydroxide
OADC: Oleic Albumin dextrose catalase
BCG: Bacillus Calmette-Guerin
JCRC: Joint Clinical Research centre
DNA: Deoxyribonucleic Acid
MTB L4-U: *Mycobacterium tuberculosis* Uganda family
MTB L4-NU: *Mycobacterium tuberculosis* lineage 4 strains other than MTB Uganda family
MTB L4: *Mycobacterium tuberculosis* lineage 4 (Euro-American lineage)
MTB L3: *Mycobacterium tuberculosis* lineage 3 (East Africa India or Central Asian Stains)
LRPS: LightCycler real-time PCR single nucleotide polymorphism assay
