# Supplementary material for: A single-nucleotide-polymorphism real-time PCR assay for genotyping of Mycobacterium tuberculosis complex in peri-urban Kampala
Source: BMC Infect Dis. 2015 Sep 30;15:396. doi: 10.1186/s12879-015-1121-7 (PMC4590274; doi:10.1186/s12879-015-1121-7)
Supplement: Additional file 1: Table S1. — Lineage-specific SNPs for MTB Uganda family, MTB lineage 4 and MTB lineage 3. (DOCX 20 kb) [file 12879_2015_1121_MOESM1_ESM.docx]

**Additional file 1: Table S1: Lineage-specific SNPs for MTB Uganda family, MTB lineage 4 and MTB lineage 3**

| **Gene (ORF)** | **SNP position** | **Wild type (H37Rv) codon** | ***Mutant codon** | **Lineage/ sub lineage** |
| --- | --- | --- | --- | --- |
| Rv004c | 619 | **G**ac | **A**ac | MTB Uganda family (MTB L4-U) |
| Rv2949c | 375 | tt**C** | tt**T** | MTB Uganda family (MTB L4-U) |
| Rv0006c | 238 | **A**cc | **G**cc | MTB Uganda family (MTB L4-U) |
| Rv2962 | 711 | gc**T** | gc**C** | MTB lineage 4 (MTB L4-NU) |
| Rv0407 | 960 | tt**T** | tt**C** | MTB lineage 4 (MTB L4-NU) |
| Rv0129c | 472 | **G**gc | **A**gc | MTB Lineage 3 (MTB L3) |
| Rv2959c | 219 | ga**G** | ga**A** | MTB Lineage 3 (MTB L3) |
| Rv3133c | 419 | gCc | gGc | MTB Lineage 3 (MTB L3) |

*Bold and capital letter shows the lineage-specific SNP
