# Supplementary material for: A single-nucleotide-polymorphism real-time PCR assay for genotyping of Mycobacterium tuberculosis complex in peri-urban Kampala
Source: BMC Infect Dis. 2015 Sep 30;15:396. doi: 10.1186/s12879-015-1121-7 (PMC4590274; doi:10.1186/s12879-015-1121-7)
Supplement: Additional file 2: Table S2. — Primer/probe used in genotyping MTB. (DOCX 21 kb) [file 12879_2015_1121_MOESM2_ESM.docx]

**Additional file 2: Table S2: Primer/probe used in genotyping MTB**

| **Name of H37Rv ORF** | **Primers/probes** | **Calculated SNP Tm /ºC** | **score** |
| --- | --- | --- | --- |
| Rv2949c^a^ | F:5-TTATAACAAGGTTGACGCAGACA-3;  R:5-CTCTCTTTCGGGAATTCTGATAC-3 |  | 62 |
|  | Probe1: ACC TTC AAG GAA GAG GCT AAA GTC T-Fluorescein  Probe2: LC Red 640-GTC GGA GAT TTG CCA GGT TGG CT-Phosphate | 59 |  |
| Rv0006^a^ | F: 5-TGCTCTATGCAATGTTCGATTC-3; R: 5-GGCTTCGGTGTACCTCA-3 |  | 23921 |
|  | Probe1: LC Red 640-GCCCATGGCCTCGGC-Phosphate  Probe2: GTCGCCGTGCGGGTGGTAG-Fluorescein | 57 |  |
| Rv004c^a^ | F:5-ATTGCTCGCATGGCAGA-3;  R: 5-AAACCAGGTACTTGTCGG-3 |  | 1626 |
|  | Probe1: LC Red 640-TGATGACGGAAAGCCGTCGAAA-Phosphate  Probe2: GTTTTCGCGGTAGGTGCCCTCGATG-Fluorescein | 58 |  |
| Rv0407^b^ | F:5-TCGACGACCCGATCGAG-3;  R:5-CTGGGTGCCAGGTCCGA-3 |  | 3130237 |
|  |  |  |  |
|  | Probe1: ACCACCTGGTATTTCACGCACCAGGAC-Fluorescein  Probe2: LC Red 610-GACCAGCGCCGGTTCCTG-Phosphate | 62 |  |
| Rv2962c^b^ | F:5-GAACGCCCTTTGCTCTTC-3;  R:5-CAAGGTACTCGTGGTTGG-3 |  | 280 |
|  | Probe1: CACACCCTGTATGCCGACG-Fluorescein  Probe 2: LC Red 610-CCCGAGCTGATGCCCACCTACGA-Phosphate | 55 |  |
| Rv0129c^c^ | F:5-CGACTGGTATCAGCCCTC-3;  R: 5-GGAACTGCTGCGGGTAGTA-3 |  | 610 |
|  | Probe 1: LC Red 610-GACACGCCCTTGTTGGCC-Phosphate  Probe 2: CGCCGCGTTGCCTGTCG-Fluorescein | 58 |  |
| Rv2959c^c^ | F: 5-TGGAAATCCGTCAGCGATA-3; R: 5-GTCCACGGTAAGTACCTTG-3 |  | 238 |
|  | Probe1: LC Red 610-TCGCTCAGGATCTCCTGGTAATTCC-Phosphate  Probe2:GAACTCGATGACGAGGGATGGTTGCA-Fluorescein | 60 |  |
| Rv3133^c^ | F: 5-GCGCCGTCAAAGATGTG-3;  R: 5-TTGTTGGTCAGGCCCTC-3 |  | 1985 |
|  | Probe 1: CGGTGCCGCCGAGAAG- Fluorescein  Probe 2: LC Red 610-GGACCCGCTATCAGGCCTTACCGA-Phosphate | 53 |  |

**^a^** Primer / probe set for MTB Uganda family; ^b^ Primer/probe set for MTB lineage 4; ^c^ Primer/probe set for MTB lineage 3. F: Forward primer, R: Reverse primer. LC Red 610/LC Red 640 attached to the oligonucleotide sequences refers to the dyes (Red 610 and Red 640) used and the channels wavelength (498-610nm and 498-640nm). The score value was automatically generated by the software: the softwares’ in-built algorithm penalizes the probe/primer set designed as it violates the set criteria for selecting probes; a probe that satisfies all the set criteria is assigned a score of “ZERO” i.e. the smaller the score value the better the probe/primer set. For instance considering “**^a^** Primer / probe set”, Rv2949c set had the lowest score (closer to zero), but we chose Rv004c because in the optimization procedures a lower concentration of the Rv004c probes was required thus reducing on the cost of the assay, Rv0006 set had the worst score and indeed no application was observed with this set. For the case of ^b^ Primer/probe set, Rv2962 set had a smaller score, so it was selected for use in the assay; for ^c^ Primer/probe set Rv2959c and Rv3133c probe/primer sets results were conflicting with positive (CAS strain) negative controls (H37Rv) so they were excluded.
