# Supplementary material for: A single-nucleotide-polymorphism real-time PCR assay for genotyping of Mycobacterium tuberculosis complex in peri-urban Kampala
Source: BMC Infect Dis. 2015 Sep 30;15:396. doi: 10.1186/s12879-015-1121-7 (PMC4590274; doi:10.1186/s12879-015-1121-7)
Supplement: Additional file 3: Table S3. — LSP-PCR and LRPS sample analyses. (DOC 43 kb) [file 12879_2015_1121_MOESM3_ESM.doc]

**Additional file 3: Table S3: LSP-PCR and LRPS sample analyses**

| **MTB lineage** | **LSP-PCR (RD 724 analysis)^a^** | **LRPS^b^** |
| --- | --- | --- |
| MTBC Uganda | 37 | 40 |
| MTBC Non-Uganda | 21 | 30 |
| No LSP-product | 12 | - |
| **Total** | **70** | **70** |
|  | | |
| **MTB lineage** | **LSP-PCR (RD 750 analysis)** | **LRPS** |
| MTBC lineage 3 | 10 | 10 |
| Non-MTBC lineage 3 | 23 | 23 |
| **Total** | **33** | **33** |

1. number of samples analyzed by LSP-PCR,
2. number of samples analyzed by LRPS
