# Supplementary figures and images for: A single-nucleotide-polymorphism real-time PCR assay for genotyping of Mycobacterium tuberculosis complex in peri-urban Kampala
Source: BMC Infect Dis. 2015 Sep 30;15:396. doi: 10.1186/s12879-015-1121-7 (PMC4590274; doi:10.1186/s12879-015-1121-7)

**Figure S1**


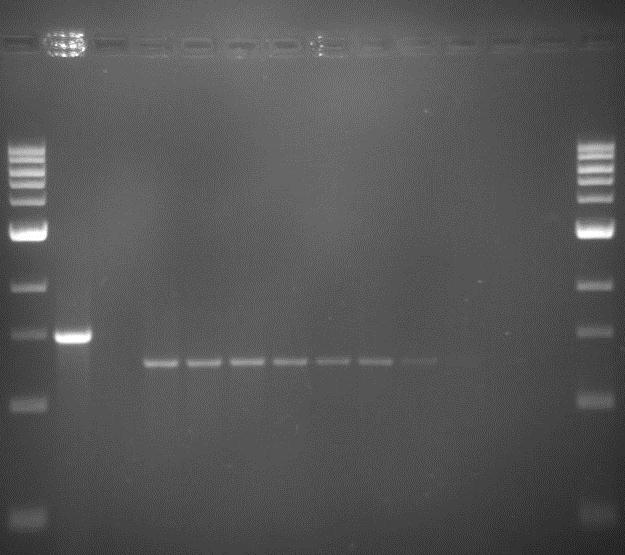

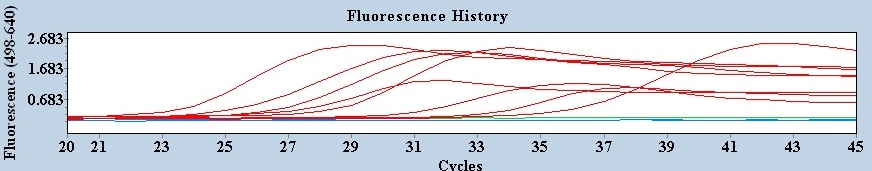


M 1 2 3 4 5 6 7 8 9 10 11 12 M

**0.5kb**

**1kb**

**1.5kb**

**(a) (b)**

**M 1 2 3 4 5 6 7 8 9 10 11 12 M**


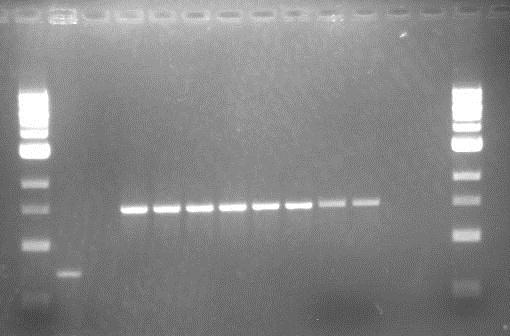

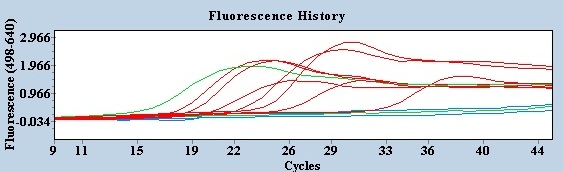


**0.5kb**

**1kb**

**1.5kb**

**(c) (d)**

Supplement: Additional file 4: Figure S1. — LRPS is more sensitive than LSP-PCR typing: H37Rv genomic DNA was serially diluted and analyzed with RD 724 (N = 10) or RD 750 (N = 10) deletion primers: lane M = molecular weight markers, lane 1 positive control (MTB Uganda family or lineage 3 strain), lane 2 negative control, lane 3- 12 H37Rv DNA diluted from 100-10 ng; the minimum dilution of genomic DNA that can be amplified was in lane 10 (30ng) (Plate a & c). Plate b & d are the corresponding LRPS using Rv004c/probe or Rv0129c/probe set with H37Rv genomic DNA diluted from (10-1) ng per assay, the minimum dilution of DNA that can be amplified was 3ng (See arrow on the amplification curves plate b & d). (DOC 179 kb) [file 12879_2015_1121_MOESM4_ESM.doc]

**Figure S2**


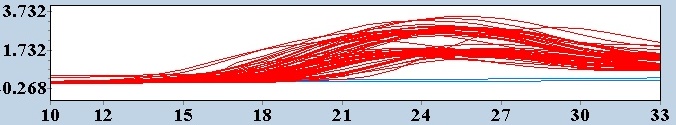


**Fluorescence (498-640) nm**

**(a)**


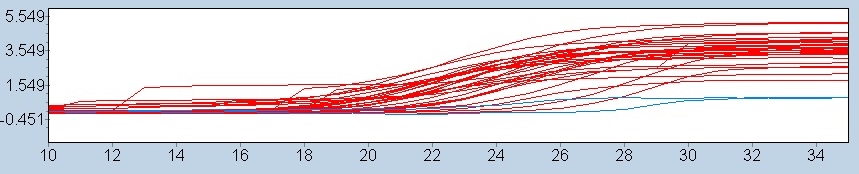


**(b)**

**Number of cycles**

Supplement: Additional file 5: Figure S2. — Fluorescence applification curve readings: Specific Rv004c and Rv0129c primer/probe set containing lineage specific SNP were used to analyze 70 and 33 MTBC isolates respectively. Amplification fluorescence curves for Rv004c and Rv0129 target region are shown in panel (a) and panel (b) respectively. (DOC 123 kb) [file 12879_2015_1121_MOESM5_ESM.doc]
